# Tailoring Functional Terminals on Solution-Processable Fullerene Electron Transporting Materials for High Performance Perovskite Solar Cells

**DOI:** 10.3390/nano12071046

**Published:** 2022-03-23

**Authors:** Fu Liu, Zhou Xing, Ya Ren, Rong-Jiao Huang, Piao-Yang Xu, Fang-Fang Xie, Shu-Hui Li, Xinxian Zhong

**Affiliations:** 1 State Key Laboratory for Chemistry and Molecular Engineering of Medicinal Resources, School of Chemistry and Pharmaceutical Sciences, Guangxi Normal University, Guilin 541004, China; liuf9612@163.com (F.L.); renya2022@163.com (Y.R.); huangrongjiao125@163.com (R.-J.H.); zhongxx2004@163.com (X.Z.); 2 Guangdong Provincial Key Laboratory of Nano-Micro Materials Research, School of Chemical Biology and Biotechnology, Shenzhen Graduate School, Peking University, Shenzhen 518055, China; 3 State Key Laboratory for Physical Chemistry of Solid Surfaces, iChEM (Collaborative Innovation Center of Chemistry for Energy Materials), Department of Chemistry, College of Chemistry and Chemical Engineering, Xiamen University, Xiamen 361005, China; 20170155073@xmu.edu.cn (P.-Y.X.); fangfangxie0707@163.com (F.-F.X.)

**Keywords:** fullerene, perovskite solar cell, electron transporting material, stability

## Abstract

Widely known as an excellent electron transporting material (ETM), pristine fullerene C_60_ plays a critical role in improving the photovoltaic performance of inverted structure perovskite solar cells (PSCs). However, the imperfect perovskite/C_60_ interface significantly limits the promotion of device performance and stability due to the weak coordination interactions between bare carbon cages and perovskite. Here, we designed and synthesized three functionalized fulleropyrrolidine ETMs (abbreviated as CEP, CEPE, and CECB), each of which was modified with the same primary terminal (cyanoethyl) and various secondary terminals (phenyl, phenethyl, and chlorobutyl). The resulting CECB-based PSC has a power conversion efficiency (PCE) over 19% and exceptional photo-stability over 1800 h. This work provides significant insight into the targeted terminal design of novel fullerene ETMs for efficient and stable PSCs.

## 1. Introduction

Perovskite solar cells (PSCs), which have emerged as a possible contender for next-generation photovoltaic technology, have shown an enormous increase in PCE over last decade [1]. PSCs can be classified into two distinct structures based on the order of hole transporting layers and electron transporting layers (ETLs), namely the regular structure and the inverted structure. Though regular structure PSCs have had the highest performance to date [2], inverted structure PSCs have attracted growing attentions due to their stable and reliable performance [3] as well as their practicability in high-efficiency tandem solar cells [4]. Nevertheless, the Shockley-Queisser (S-Q) limit determines the maximum PCE for both types of devices. The S-Q limit is calculated using the electrical energy extracted by incident photons [5]. To improve the PCE toward the S-Q limit, one needs to focus on mitigating various energy losses in solar cells, including the coherent absorption-emission effect, Carnot loss, Boltzmann loss, thermalization loss, and in-band loss [6]. Thermalization loss caused by heat dissipation of hot carriers and in-band loss within the perovskite absorber are the two major loss pathways, accounting for over 50% of the total energy losses. Although various great works have verified that external light spectral tuning methodologies could manage the aforementioned two dominant losses [7], manufacturing of complicated extra photonic/thermal accessories can be a noticeable problem for large-scale device production. Thus, seeking the opportunity of intrinsically optimizing materials (e.g., perovskite and charge transporting materials [8,9], plasmonic nanomaterials [10,11], and up-conversion materials [6]) and devices (interfacial charge extraction/transport/collection [12]) stimulates enormous interest among diverse communities.

The perovskite/fullerene heterojunction has a significant effect on the performance and stability of inverted structure PSCs. Fullerene C_60_, a well-known as excellent ETM, shows many charming characteristics (e.g., effective electron extraction ability, high electron mobility, well-regulated energy levels, etc.) [13,14,15], which contribute to a nano-level interfacial contact for improved charge extraction and a strong humidity-resistance for improved protection of the bottom perovskite films, resulting in suppressed hysteresis and enhanced stability of the devices [3,16]. However, further optimization of the device is limited by the weak coordination interaction between bare carbon cages and perovskite [17]. As a result, to strengthen the intrinsic chemical bond between perovskite and fullerene heterojunction, researchers modified fullerene cages with various functional terminals such as nitrogen-containing groups, oxygen-containing groups, and sulfur-containing groups [18,19], all of which can specifically interact with the perovskite material.

However, defects at the surface or grain boundaries of polycrystalline perovskite films, also known as non-radiative recombination sites, accelerate the structural decomposition of perovskite, causing device instability and ultimately device failure [20,21,22,23,24]. In principle, negatively charged deep traps induced by Pb-I antisite defects or uncoordinated halide ions can be efficiently passivated by fullerene cages [25,26], whereas positively charged Pb-related defects cannot. In this way, it’s demonstrated that the introduction of electron-donating molecules can be an effective strategy to passivate the corresponding defects via Lewis base-acid interaction between them [27]. Hence, one needs to adopt appropriate strategies to install coordinated or passivated groups onto the convex of fullerene cages with the purpose of optimizing the device’s performance and stability

In contrast to the complicated synthesis (e.g., multi-step reaction under high temperature or light illumination) of the most prevalent solution-processable fullerene ETM, [6,6]-phenyl-C61-butyric acid methyl ester, we deliberately designed and synthesized three functional fulleropyrrolidine derivatives (abbr. CEP, CEPE, and CECB, respectively) via the simple Prato reaction, and then applied them as ETMs in inverted structure PSCs. The synthesized fulleropyrrolidine derivatives are composed of three ingredients designed within them: a C_60_ body with a strong electron transporting ability, a primary terminal for enhancing solubility, and a secondary terminal with a coordination interaction with perovskite. As a result, we found that the CECB afforded the best PCE exceeding 19%, showing a T_80_ lifetime (80% initial PCE) for over 1800 h which is one of the highest values among fulleropyrrolidine-based PSCs in the reported literature. Additionally, we summarized the effects of different functional terminals with respect to steric hindrance and rigidity on the photovoltaic parameters of devices, which could provide insight for the molecular design of efficient fullerene ETMs in the future.

## 2. Materials and Methods

### 2.1. Materials

The indium tin oxide (ITO) glasses were supplied by Advanced Election Technology Co., Ltd. (Liaoning, China). The poly[bis(4-phenyl)(2,4,6-trimethylphenyl)amine (PTAA), bathocuproine (BCP) and raw materials for perovskite were supplied by Xi’an Polymer Light Technology Corp (Xi’an, China). C_60_ was purchased from Puyang Yongxin Fullerene Co., Ltd (Henan, China). Other materials or solvents were all supplied by Sigma-Aldrich (St. Louis, MO, USA) or Alfa-Aesar (Haverhill, MA, USA) and used as received.

### 2.2. Device Fabrication

ITO glasses were cleaned in an ultrasonic bath with detergent, deionized water, acetone, and isopropanol for 15 min and then dried with N_2_. Before use, the substrates were treated by with UV-ozone for 10 min. The devices were fabricated in a nitrogen glovebox, in which the content of water and oxygen was below 1 ppm. First, 2 mg mL^−1^ PTAA in toluene was spin-coated at 4000 rpm for 30 s, and then annealed at 100 °C for 10 min. The perovskite [Cs_0.05_(FA_0.95_MA_0.05_)_0.95_Pb(I_0.95_Br_0.05_)_3_] film was fabricated according to previous work [28]. In a two-step method, 70 μL of perovskite precursor solution was dripped on the substrate and spin-coated at 1300 rpm for 10 s and 5000 rpm for 20 s. At the last 5 s of the second step, 150 μL chlorobenzene was added as an anti-solvent to enhance the crystallization of the perovskite film. Subsequently, all fullerenes in this work were completely dissolved in *o*-dichlorobenzene (*o*-DCB) with a concentration of 20 mg mL^−1^, and then spin-coated at 3000 rpm for 20 s. The spin-coating of 0.5 mg mL^−1^ BCP in isopropanol at 6000 rpm for 30 s was followed by 10 min annealing at 80 °C. Finally, 70 nm Ag electrode was thermally evaporated at 2 × 10^−4^ Pa to finish device fabrication.

### 2.3. Characterization

The NMR spectra were measured on the Bruker Biospin Advance III at 500 MHz (Billerica, MA, USA) or the Bruker Avance Neo at 600 MHz (Billerica, MA, USA) with CDCl_3_ as the internal lock. The mass spectra were recorded on a Bruker Esquire HCT mass spectrometer (Billerica, MA, USA) with an atmospheric pressure chemical ionization ion source. The cyclic voltammetry (CV) tests were conducted using a Shanghai Chenhua CHI-660E electrochemical workstation (Shanghai, China). The UV-Vis absorption spectra were measured on a Cary 60 spectrometer (Agilent, Santa Clara, CA, USA). Photovoltaic performances of devices were characterized under AM 1.5 G 100 mW cm^−2^ by using a 300 W xenon solar simulator (Newport Oriel Solar Simulators, Deere Ave, Irvine, CA, USA) with an aperture of 0.06 cm^2^. Current density-voltage (*J*-*V*) curves were recorded by using a Keithley 2400 source meter (Cleveland, OH, USA). External quantum efficiency (EQE) was tested by using a Merlin lock-in amplifier coupled with a CS260 monochromator and a 300 W xenon lamp (Newport, Deere Ave, Irvine, CA, USA). Steady-state output at maximum power point (MPP) was recorded on a CHI-660E electrochemical workstation (Shanghai, China) under light illumination. Light-soaking stability was performed at room temperature (RT) with a white-LED light under N_2_ atmosphere. Electron mobilities were determined by fabricating electron-only devices with a configuration of ITO/TiO_x_/fullerene/TiO_x_/Ag according to our previous work [29]. Steady-state photoluminescence spectra (PL) were measured by a Hitachi F7000 Fluorescence Spectrophotometer (Tokyo, Japan) with an excitation wavelength of 516 nm. The cross-sectional image of the corresponding device was obtained by a Hitachi S-4800 scanning electron microscope (SEM).

## 3. Results and Discussion

### 3.1. Synthesis and Characterization of Fullerene Compounds

**Figure 1:**
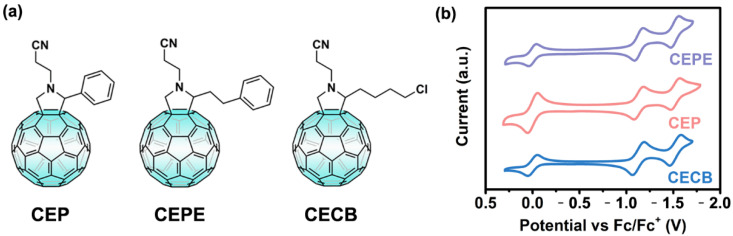
(**a**) Chemical structures and (**b**) CV curves of CEP, CEPE and CECB in a mixed solution of *o*-DCB/acetonitrile (5:1, *v*/*v*) with 0.1 M Bu_4_NPF_6_ at a scan rate of 100 mV/s. Fc/Fc^+^ was used as the internal standard.

We designed and synthesized three fulleropyrrolidine derivatives, namely *N*-(2-cyanoethyl)-2-phenyl [60]fulleropyrrolidine (CEP), *N*-(2-cyanoethyl)-2-phenethyl [60]fulleropyrrolidine (CEPE), and *N*-(2-cyanoethyl)-2-(4-chlorobutyl) [60]fulleropyrrolidine (CECB), respectively, as shown in Figure 1a. The synthetic routes and purification methods were described in Schemes S1–S3 in the Supporting Information [30]. All chemical structures were confirmed by ^1^H NMR, ^13^C NMR, and mass spectrometry (Figures S1–S8). Subsequently, we managed to elucidate the coordination interaction between designed terminals and perovskite components by chemical shifts in NMR spectra as shown in Figures S9–S11 [23,31,32]. Generally, all three compounds were similarly modified with cyanoethyl groups, performing a synergic role as a solubility enhancer for the fullerene cage and a Lewis-base terminal to coordinate with the Pb^2+^ ion (Figure S9). Presented in Figure S10 is the NMR evidence for supramolecular interactions contributed from the benzene rings [32]. Additionally, benzene moieties in CEP and CEPE served as the *π*-*π* interaction inducer to promote the orderly arrangement of fulleropyrrolidine molecules [30]. The only difference between the two fullerenes is the distance from pyrrolidine moiety to benzene ring, which might give rise to different packing modes of fullerene cages. Likewise, targeted on passivating the uncoordinated Pb^2+^ ions in perovskite, compound CECB was decorated by a chlorine atom at the end of the butyl chain with an enhanced flexibility of terminal in comparison to CEP and CEPE [25].

To investigate the potential of three synthesized fulleropyrrolidine derivatives as ETMs for PSCs, UV-Vis absorption, and CV characterizations were performed to determine their optical and electrochemical properties, as shown in Figures S12–S14, Figure 1b, and Table S1 [33]. Obviously, all compounds showed completely reversible CV signals. Meanwhile, based on ferrocene/ferrocenium (Fc/Fc^+^) redox as the reference, the lowest unoccupied molecular orbital (LUMO) values of CEP, CEPE, and CECB were calculated to be −3.74 eV, −3.74 eV, and −3.75 eV, well matching with the conduction band minimum of perovskite for efficient electron transport.

### 3.2. Photoelectric Properties of Fullerene Films

**Figure 2:**
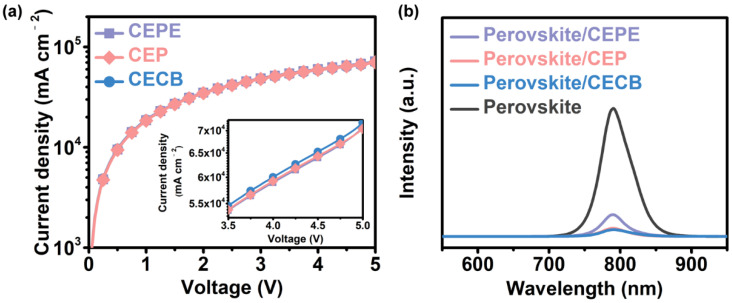
(**a**) Electron mobility measurements of different fullerene films. Inset is the detail with enlarged scale at 3.5–5.0 V. (**b**) Steady-state PL spectra of different fullerene films deposited on the perovskite layers.

Since the photovoltaic performance of a device significantly hinges on the type of fullerene ETL, one needs to pay attention to the photoelectric properties of fullerene films [34]. In view of the different chemical structures and electrochemical properties of CEPE, CEP, and CECB as discussed above, it is reasonable to deduce that they would afford diverse properties in the film state and therefore affect the photovoltaic performance of the device [30]. To verify our speculation, we performed electron mobility measurements of different fullerene films based on the electron-only devices as shown in Figure 2a. The calculated electron mobilities of CEPE, CEP, and CECB were 4.0 × 10^−4^, 4.6 × 10^−4^, and 4.9 × 10^−4^ cm^2^V^−1^ s^−1^, respectively, showing that the CECB ETL might demonstrate the best electron transporting ability among these three samples. 

Later on, we further recorded steady-state PL spectra of different fullerene films deposited on the perovskite films. The PL quenching situation showing that all three fullerene compounds can be good ETMs for the PSCs is shown in Figure 2b. In particular, the CECB exhibited the best PL quenching ability, followed by CEP and then CEPE, indicating the superiority of the CECB, which was consistent with the electron mobility. We attributed these differences in photoelectric properties to the different fullerene structures with respect to the lengths and steric hindrance of side chains on the convex of fullerene cages [30,35].

### 3.3. Photovoltaic Performance of Fullerene-Based Devices

**Figure 3:**
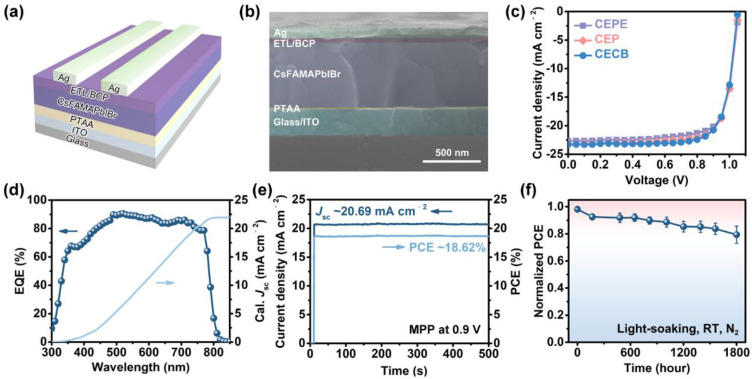
(**a**) Diagram of device structure. (**b**) Cross-sectional SEM image of the device. (**c**) *J-V* curves of champion devices based on different fullerene ETMs. (**d**) EQE and calculated *J*_sc_ curves of the champion CECB-based device. (**e**) Steady-state output test of the champion CECB-based device at MPP. (**f**) Light-soaking stability of the champion CECB-based device.

To evaluate the photovoltaic performance of these fullerene compounds when used as ETMs in devices, we fabricated inverted structure PSCs with the configuration ITO/PTAA/perovskite/ETL/BCP/Ag as shown in Figure 3a. The cross-sectional SEM image of the corresponding device with specific functional layer thicknesses, e.g., ITO (180 nm), PTAA (20 nm), perovskite (550 nm), fullerene/BCP (30 nm), and Ag (70 nm), is shown in Figure 3b. We tested the *J-V* curves of devices with various fullerene ETMs after photo-activating them for 5 min in ambient air. The CECB-based PSC achieved the highest PCE of 19.05% among these three fullerene ETMs, while the CEP-based PSC showed a modest PCE of 18.68%, and the CEPE-based PSC possessed the lowest PCE of 18.33%, respectively (Figure 3c). EQE measurement (Figure 3d) and negligible hysteresis in different scan directions (Figure S15) confirmed the accuracy of the measured *J*_sc_ value for the optimal CECB-based PSC. 

Additionally, we performed steady-state output measurements at MPP to evaluate the reliability of the CECB-based PSC under operating conditions. The champion device showed negligible damping of the *J*_sc_ value during the light-soaking process within 500 s, as shown in Figure 3e. Furthermore, we continued to measure the long-term light-soaking stability of the CECB-based devices at RT under continuous white-LED illumination in N_2_ atmosphere. As presented in Figure 3f, the CECB-based devices (average for 10 devices) showed a T_80_ lifetime of over 1800 h, showing out the excellent photo-stability of the CECB ETM. In addition, the obtained lifetime is one of the longest for fulleropyrrolidine-based PSCs to date (Table S2) [18,30,36,37,38,39,40,41,42,43], which is attributed to the functional terminals introduced.

### 3.4. Side-Chain Effects of Fullerenes on Photovoltaic Parameters of Devices

**Figure 4:**
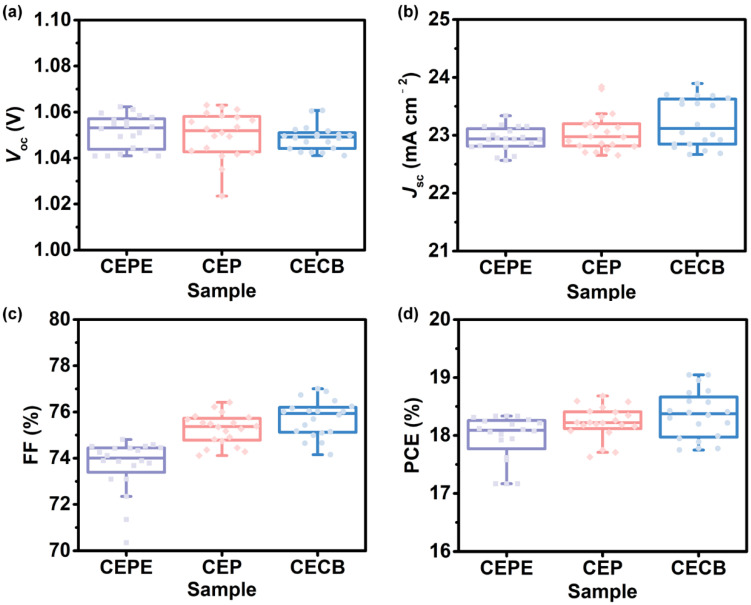
Photovoltaic performance of 20 devices based on different fullerene ETMs: (**a**) *V*_oc_, (**b**) *J*_sc_, (**c**) FF, and (**d**) PCE.

To further compare the photovoltaic parameters, we fabricated 20 devices for each fullerene ETM. The statistic diagrams of *V*_oc_, *J*_sc_, fill factor (FF), and PCE are shown in Figure 4 and Table S3. We considered that the almost same LUMO energy level of these three fullerene ETMs led to a similar *V*_oc_ value of the device, as shown in Figure 4a. As for *J*_sc_ values, the CECB film possessed the highest electron mobility, resulting in the largest *J*_sc_ value contributed by the excellent electron transporting ability [44].

Interestingly, we found that the FF value can be the major issue affecting the ultimate PCE of devices. It is obvious that the CECB-based devices showed a higher average FF value than that of CEP and CEPE. Basing on our previous work upon the relationships between fullerene structure and device performance, bulky pendent groups with sizable steric hindrance or too much long side chains would be harmful to the compact packing of fullerene cages, resulting in lower electron mobility of the fullerene film, which in turn, negatively affects the photovoltaic performance of the resultant device [30,35]. In this work, if we set the CEP as a model molecule for comparison, the cyanoethyl group ensured a good solubility for the fullerene cage, and the rigid phenyl group might be located at the interval of neighboring fullerene cages, tending to separate the neighboring fullerenes. When compared with the CEPE, the longer alkyl chain contributes to better solubility for the fullerene cage [45]. However, it magnified the steric hindrance of the phenyl-based side-chain, resulting in an alienated packing mode of fullerene cages, which increased the energy barrier for electrons transporting among the delocalized *π*-*π* system of fullerenes [30].

As for the CECB, the longer and more flexible alkyl chain further increases the solubility of the fullerene cage, resulting in a better film morphology and thus optimal interfacial contact [34]. However, in comparison to the benzene ring, we considered that the flexible alkyl chain with a smaller chlorine atom at the terminal would wrap around or locate at the interval of neighboring fullerene cages without any negative effect on molecular packing, which is beneficial for electron transport. Both of the aforementioned issues have a direct influence on the series resistance (*R*_s_) and shunt resistance (*R*_sh_) (Table S3), and finally determine the FF value of devices [46,47,48]. Finally, the direct comparison of these three fullerene compounds reveals the side-chain effects of the fullerene ETMs on the photovoltaic parameters of the ultimate devices, which is crucial for the molecular design of functionalized fullerene materials toward high-performance and stable PSCs with fewer interfacial energy losses. 

## 4. Conclusions

Fullerenes have been demonstrated to be the irreplaceable ETMs for inverted structure PSCs. In this work, we successfully synthesized three functionalized fullerene ETMs via the facile Prato method, confirming the reliability and superiority of the CECB from the molecular level to the film state, and finally the device level. The champion device achieved a PCE over 19% and simultaneously showed excellent stability for over 1800 h under continuous light illumination, which is one of the highest values for fulleropyrrolidine-based PSCs as of now. Furthermore, we discussed the side-chain effects, including length, rigidity, and steric hindrance, on the photovoltaic performance of fullerene ETMs as well as the photovoltaic parameters of devices, demonstrating the importance of molecular design with regard to suitable side chains on the convex of fullerene cages. We believe our results give scientific insight on targeted design for the one who wants to exploit novel fullerene ETMs in the future.

## Data Availability

The data are available on reasonable request from the corresponding author.

## Supplementary Materials

The following supporting information can be downloaded at: https://www.mdpi.com/article/10.3390/nano12071046/s1, Scheme S1. Synthesis of the compound CEP; Scheme S2: Synthesis of the compound CEPE; Scheme S3: Synthesis of the compound CECB; Figure S1: 1H NMR spectrum (600 MHz) of compound CEP; Figure S2: 13C NMR spectrum (150 MHz) of compound CEP; Figure S3: 1H NMR spectrum (600 MHz) of compound CEPE; Figure S4: 13C NMR spectrum (150 MHz) of compound CEPE; Figure S5: APCI-MS spectrum of compound CEPE; Figure S6: 1H NMR spectrum (500 MHz) of compound CECB; Figure S7: 13C NMR spectrum (125 MHz) of compound CECB; Figure S8: APCI-MS spectrum of compound CECB; Figure S9: The comparison of NMR spectra between MeCN and mixture of MeCN + PbI2 in DMSO-*d*_6_ contains 0.03% (*v*/*v*) TMS; Figure S10: The comparison of NMR spectra between PhCH3 and mixture of PhCH3 + PbI2 in DMSO-*d*_6_ contains 0.03% (*v*/*v*) TMS; Figure S11: The comparison of NMR spectra between *n*-BuCl and mixture of *n*-BuCl + PbI2 in DMSO-*d*_6_ contains 0.03% (*v*/*v*) TMS; Figure S12: UV-Vis absorption spectra of CEP in toluene solution; Figure S13: UV-Vis absorption spectra of CEPE in toluene solution; Figure S14: UV-Vis absorption spectra of CECB in toluene solution; Figure S15: *J-V* curves of CECB-based device under different scan directions; Table S1: Electrochemical and optical properties of CEP, CEPE and CECB; Table S2: Photovoltaic performance and stability of fulleropyrrolidine-based PSCs in the literature; Table S3: Photovoltaic performance of 20 devices based on different fullerene ETMs.

## References

[1] NREL. https://www.nrel.gov/pv/assets/pdfs/cell-pv-eff-emergingpv-rev211214.pdf.

[2] Kim M., Jeong J., Lu H., Lee Tae K., Eickemeyer Felix T., Liu Y., Choi In W., Choi Seung J., Jo Y., Kim H.-B. (2022). Conformal quantum dot–SnO_2_ layers as electron transporters for efficient perovskite solar cells. Science.

[3] Chen B., Rudd P.N., Yang S., Yuan Y., Huang J. (2019). Imperfections and their passivation in halide perovskite solar cells. Chem. Soc. Rev..

[4] Lin R., Xu J., Wei M., Wang Y., Qin Z., Liu Z., Wu J., Xiao K., Chen B., Park S.M. (2022). All-perovskite tandem solar cells with improved grain surface passivation. Nature.

[5] Shockley W., Queisser H.J. (1961). Detailed balance limit of efficiency of *p*-*n* junction solar cells. J. Appl. Phys..

[6] Wang K., Zheng L., Hou Y., Nozariasbmarz A., Poudel B., Yoon J., Ye T., Yang D., Pogrebnyakov A.V., Gopalan V. (2022). Overcoming Shockley-Queisser limit using halide perovskite platform?. Joule.

[7] Zhou Z., Chen Q., Bermel P. (2015). Prospects for high-performance thermophotovoltaic conversion efficiencies exceeding the Shockley–Queisser limit. Energ. Convers. Manag..

[8] Castro E., Murillo J., Fernandez-Delgado O., Echegoyen L. (2018). Progress in fullerene-based hybrid perovskite solar cells. J. Mater. Chem. C.

[9] Liang Y., Song P., Tian H., Tian C., Tian W., Nan Z., Cai Y., Yang P., Sun C., Chen J. (2021). Lead leakage preventable fullerene-porphyrin dyad for efficient and stable perovskite solar cells. Adv. Funct. Mater..

[10] Laska M., Krzemińska Z., Kluczyk-Korch K., Schaadt D., Popko E., Jacak W.A., Jacak J.E. (2020). Metallization of solar cells, exciton channel of plasmon photovoltaic effect in perovskite cells. Nano Energy.

[11] Jacak W.A., Jacak J.E. (2019). New channel of plasmon photovoltaic effect in metalized perovskite solar cells. J. Phys. Chem. C.

[12] Li Y., Xie H., Lim E.L., Hagfeldt A., Bi D. (2022). Recent progress of critical interface engineering for highly efficient and stable perovskite solar cells. Adv. Energy Mater..

[13] Fang Y., Bi C., Wang D., Huang J. (2017). The functions of fullerenes in hybrid perovskite solar cells. ACS Energy Lett..

[14] Deng L.-L., Xie S.-Y., Gao F. (2018). Fullerene-based materials for photovoltaic applications: Toward efficient, hysteresis-free, and stable perovskite solar cells. Adv. Electron. Mater..

[15] Jia L., Chen M., Yang S. (2020). Functionalization of fullerene materials toward applications in perovskite solar cells. Mater. Chem. Front..

[16] Tian C., Sun C., Chen J., Song P., Hou E., Xu P., Liang Y., Yang P., Luo J., Xie L. (2022). Fullerene derivative with flexible alkyl chain for efficient tin-based perovskite solar cells. Nanomaterials.

[17] Warby J., Zu F., Zeiske S., Gutierrez-Partida E., Frohloff L., Kahmann S., Frohna K., Mosconi E., Radicchi E., Lang F. (2022). Understanding performance limiting interfacial recombination in pin perovskite solar cells. Adv. Energy Mater..

[18] Li B., Zhen J., Wan Y., Lei X., Liu Q., Liu Y., Jia L., Wu X., Zeng H., Zhang W. (2018). Anchoring fullerene onto perovskite film via grafting pyridine toward enhanced electron transport in high-efficiency solar cells. ACS Appl. Mater. Interfaces.

[19] Castro E., Fernandez-Delgado O., Arslan F., Zavala G., Yang T., Seetharaman S., D’Souza F., Echegoyen L. (2018). New thiophene-based C_60_ fullerene derivatives as efficient electron transporting materials for perovskite solar cells. New J. Chem..

[20] Rajagopal A., Yao K., Jen A.K.Y. (2018). Toward perovskite solar cell commercialization: A perspective and research roadmap based on interfacial engineering. Adv. Mater..

[21] Zhang H., Nazeeruddin M.K., Choy W.C.H. (2019). Perovskite photovoltaics: The significant role of ligands in film formation, passivation, and stability. Adv. Mater..

[22] Xu G., Xue R., Chen W., Zhang J., Zhang M., Chen H., Cui C., Li H., Li Y., Li Y. (2018). New strategy for two-step sequential deposition: Incorporation of hydrophilic fullerene in second precursor for high-performance p-i-n planar perovskite solar cells. Adv. Energy Mater..

[23] Tan H., Jain A., Voznyy O., Lan X., García de Arquer F.P., Fan James Z., Quintero-Bermudez R., Yuan M., Zhang B., Zhao Y. (2017). Efficient and stable solution-processed planar perovskite solar cells via contact passivation. Science.

[24] Stolterfoht M., Wolff C.M., Márquez J.A., Zhang S., Hages C.J., Rothhardt D., Albrecht S., Burn P.L., Meredith P., Unold T. (2018). Visualization and suppression of interfacial recombination for high-efficiency large-area pin perovskite solar cells. Nat. Energy.

[25] Wu B.-S., An M.-W., Chen J.-M., Xing Z., Chen Z.-C., Deng L.-L., Tian H.-R., Yun D.-Q., Xie S.-Y., Zheng L.-S. (2021). Radiation-processed perovskite solar cells with fullerene-enhanced performance and stability. Cell Rep. Phys. Sci..

[26] Wang Q., Shao Y., Dong Q., Xiao Z., Yuan Y., Huang J. (2014). Large fill-factor bilayer iodine perovskite solar cells fabricated by a low-temperature solution-process. Energy Environ. Sci..

[27] Noel N.K., Abate A., Stranks S.D., Parrott E.S., Burlakov V.M., Goriely A., Snaith H.J. (2014). Enhanced photoluminescence and solar cell performance via Lewis base passivation of organic–inorganic lead halide perovskites. ACS Nano.

[28] Li F., Deng X., Qi F., Li Z., Liu D., Shen D., Qin M., Wu S., Lin F., Jang S.-H. (2020). Regulating surface termination for efficient inverted perovskite solar cells with greater than 23% efficiency. J. Am. Chem. Soc..

[29] Xing Z., Li S.-H., Hui Y., Wu B.-S., Chen Z.-C., Yun D.-Q., Deng L.-L., Zhang M.-L., Mao B.-W., Xie S.-Y. (2020). Star-like hexakis[di(ethoxycarbonyl)methano]-C_60_ with higher electron mobility: An unexpected electron extractor interfaced in photovoltaic perovskites. Nano Energy.

[30] Xing Z., Liu F., Li S.-H., Chen Z.-C., An M.-W., Zheng S., Jen A.K.Y., Yang S. (2021). Multifunctional molecular design of a new fulleropyrrolidine electron transport material family engenders high performance of perovskite solar cells. Adv. Funct. Mater..

[31] Liu K., Dai S., Meng F., Shi J., Li Y., Wu J., Meng Q., Zhan X. (2017). Fluorinated fused nonacyclic interfacial materials for efficient and stable perovskite solar cells. J. Mater. Chem. A.

[32] Wei D., Ma F., Wang R., Dou S., Cui P., Huang H., Ji J., Jia E., Jia X., Sajid S. (2018). Ion-migration inhibition by the cation–π Interaction in perovskite materials for efficient and stable perovskite solar cells. Adv. Mater..

[33] Zhan X.-X., Zhang X., Dai S.-M., Li S.-H., Lu X.-Z., Deng L.-L., Xie S.-Y., Huang R.-B., Zheng L.-S. (2016). Tailorable PC_71_BM isomers: Using the most prevalent electron acceptor to obtain high-performance polymer solar cells. Chem. Eur. J..

[34] Jeng J.-Y., Chiang Y.-F., Lee M.-H., Peng S.-R., Guo T.-F., Chen P., Wen T.-C. (2013). CH_3_NH_3_PbI_3_ perovskite/fullerene planar-heterojunction hybrid solar cells. Adv. Mater..

[35] Xing Z., Li S.-H., Xu P.-Y., Tian H.-R., Deng L.-L., Yao Y.-R., Chen B.-W., Xie F.-F., An M.-W., Yun D.-Q. (2022). Crystallographic understanding of photoelectric properties for C_60_ derivatives applicable as electron transporting materials in perovskite solar cells. Chem. Res. Chin. Univ..

[36] Tian C., Castro E., Wang T., Betancourt-Solis G., Rodriguez G., Echegoyen L. (2016). Improved performance and stability of inverted planar perovskite solar cells using fulleropyrrolidine layers. ACS Appl. Mater. Interfaces.

[37] Li Y., Lu K., Ling X., Yuan J., Shi G., Ding G., Sun J., Shi S., Gong X., Ma W. (2016). High performance planar-heterojunction perovskite solar cells using amino-based fulleropyrrolidine as the electron transporting material. J. Mater. Chem. A.

[38] Wang Y.-C., Li X., Zhu L., Liu X., Zhang W., Fang J. (2017). Efficient and hysteresis-free perovskite solar cells based on a solution processable polar fullerene electron transport layer. Adv. Energy Mater..

[39] Castro E., Zavala G., Seetharaman S., D’Souza F., Echegoyen L. (2017). Impact of fullerene derivative isomeric purity on the performance of inverted planar perovskite solar cells. J. Mater. Chem. A.

[40] Chang J., Wang Y.-C., Song C., Zhu L., Guo Q., Fang J. (2018). Carboxylic ester-terminated fulleropyrrolidine as an efficient electron transport material for inverted perovskite solar cells. J. Mater. Chem. C.

[41] Liu X., Li P., Zhang Y., Hu X., Duan Y., Li F., Li D., Shao G., Song Y. (2019). High-efficiency perovskite solar cells based on self-assembly n-doped fullerene derivative with excellent thermal stability. J. Power Sources.

[42] Luo Z., Wu F., Zhang T., Zeng X., Xiao Y., Liu T., Zhong C., Lu X., Zhu L., Yang S. (2019). Designing a perylene diimide/fullerene hybrid as effective electron transporting material in inverted perovskite solar cells with enhanced efficiency and stability. Angew. Chem. Int. Ed..

[43] Li B., Zhen J., Wan Y., Lei X., Jia L., Wu X., Zeng H., Chen M., Wang G.-W., Yang S. (2020). Steering the electron transport properties of pyridine-functionalized fullerene derivatives in inverted perovskite solar cells: The nitrogen site matters. J. Mater. Chem. A.

[44] Liang P.-W., Chueh C.-C., Williams S.T., Jen A.K.Y. (2015). Roles of fullerene-based interlayers in enhancing the performance of organometal perovskite thin-film solar cells. Adv. Energy Mater..

[45] Gil-Escrig L., Momblona C., Sessolo M., Bolink H.J. (2016). Fullerene imposed high open-circuit voltage in efficient perovskite based solar cells. J. Mater. Chem. A.

[46] Green M.A. (1981). Solar cell fill factors: General graph and empirical expressions. Solid-State Electron..

[47] Singal C.M. (1981). Analytical expression for the series-resistance-dependent maximum power point and curve factor for solar cells. Sol. Cells.

[48] Green M.A. (1982). Accuracy of analytical expressions for solar cell fill factors. Sol. Cells.

