# Supplementary material for: Tailoring Functional Terminals on Solution-Processable Fullerene Electron Transporting Materials for High Performance Perovskite Solar Cells
Source: Nanomaterials (Basel). 2022 Mar 23;12(7):1046. doi: 10.3390/nano12071046 (PMC9000481; doi:10.3390/nano12071046)
Supplement: Supplementary file 1 [file nanomaterials-12-01046-s001.zip › nanomaterials-1626763-supplementary.pdf]

nanomaterials

Supplementary Information

Tailoring Functional Terminals on Solution-Processable Fullerene Electron Transporting Materials for High Performance Perovskite Solar Cells

Fu Liu [1], Zhou Xing [2,*], Ya Ren [1], Rong-Jiao Huang [1], Piao-Yang Xu [3], Fang-Fang Xie [3], Shu-Hui Li [1,*] and Xinxian Zhong [1]

[1] State Key Laboratory for Chemistry and Molecular Engineering of Medicinal Resources, School of Chemistry and Pharmaceutical Sciences, Guangxi Normal University, Guilin 541004, China; mailto:liuf9612@163.com (F.L.); mailto:renya2022@163.com (Y.R.); mailto:huangrongjiao125@163.com (R.-J.H.); zhongxx2004@163.com (X.Z.)
[2] Guangdong Provincial Key Laboratory of Nano-Micro Materials Research, School of Chemical Biology and Biotechnology, Shenzhen Graduate School, Peking University, Shenzhen 518055, China
[3] State Key Laboratory for Physical Chemistry of Solid Surfaces, iChEM (Collaborative Innovation Center of Chemistry for Energy Materials), Department of Chemistry, College of Chemistry and Chemical Engineering, Xiamen University, Xiamen 361005, China; 20170155073@xmu.edu.cn (P.-Y.X.); mailto:fangfangxie0707@163.com (F.-F.X.)
* Correspondence: zhouxing@pku.edu.cn (Z.X.); gxnulsh@gxnu.edu.cn (S.-H.L.)

1. Experimental Details Regarding the Synthesis and Characterization.

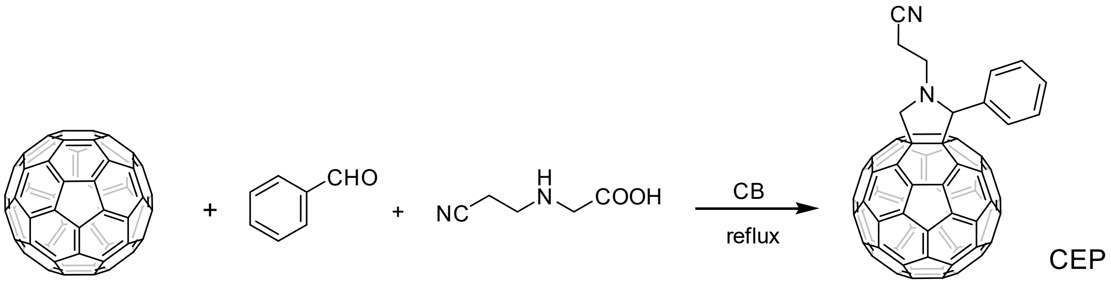

Scheme S1. Synthesis of the compound CEP.

Synthesis of compound *N*-(2-cyanoethyl)-2-phenyl [60]fulleropyrrolidine (CEP). A mixture of C_{60} (0.05 mmol, 36 mg), benzaldehyde (0.1 mmol, 10.5 μL), *N*-(2-cyanoethyl) glycine (0.2 mmol, 32 mg) was dissolved in anhydrous chlorobenzene (10 mL) under the inert gas protection. The reaction mixture was stirred at reflux for 1.5 h (detected by TLC). The crude product was concentrated in vacuo and purified on a chromatographic column using toluene as the eluent to afford the desired product of CEP. After precipitation with methanol and drying, the brown solid of CEP was obtained with an isolated yield of 41.5% (18.5 mg). Our previous work reported the compound CEP, [1] so its structure was identified only by ^{1}H and ^{13}C NMR spectra. ^{1}H NMR (600 MHz, $CS_2/CDCl_3$): δ (ppm), 7.85 (s, 2H), 7.47–7.44 (m, 2H) 7.38–7.36(m, 3H), 5.21 (s, 1H), 5.17 (d, *J* = 9.0 Hz, 1H), 4.29 (d, *J* = 9 Hz, 1H), 3.64–3.59(m, 1H), 3.02-2.96(m, 2H), 2.94–2.89(m, 1H). ^{13}C NMR (150 MHz, $CS_2/CDCl_3$): δ (ppm), 155.81, 153.60, 153.02, 152.85, 147.49, 146.73, 146.49, 146.45, 146.43, 146.39, 146.33, 146.30, 146.28, 146.11, 146.09, 146.02, 145.88, 145.76, 145.69, 145.63, 145.56, 145.47, 145.42, 145.32, 144.85, 144.74, 144.52, 144.50, 143.31, 143.13, 142.85, 142.74, 142.73, 142.72, 142.40, 142.34, 142.31, 142.30, 142.24, 142.19, 142.14, 142.13, 142.03, 142.00, 141.82, 141.65, 140.35, 140.32, 140.03, 139.60, 137.02, 136.81, 136.28, 135.95, 135.86, 129.15, 129.11, 118.62, 82.05, 76.33, 68.75, 66.65, 48.18.

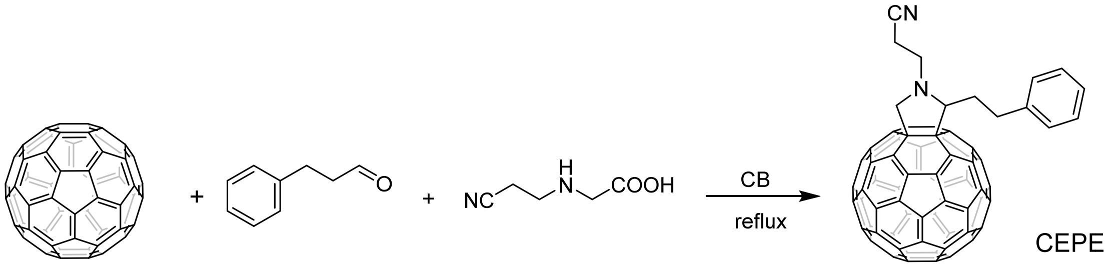

Scheme S2. Synthesis of the compound CEPE.

Synthesis of compound *N*-(2-cyanoethyl)-2-phenethyl [60]fulleropyrrolidine (CEPE). A mixture of C_{60} (0.05 mmol, 36 mg), 3-phenylpropanal (0.1 mmol, 13.0 μL), *N*-(2-cyanoethyl) glycine (0.2 mmol, 32 mg) was dissolved in anhydrous chlorobenzene (10 mL) under the inert gas protection. The reaction mixture was stirred at reflux for 1.5 h (detected by TLC). The crude product was concentrated in vacuo and purified on a chromatographic column using toluene as the eluent to afford the desired product of CEPE. After precipitation with methanol and drying, the brown solid of CEPE was obtained with an isolated yield of 38.1% (17.5 mg). ^{1}H NMR (600 MHz, $CDCl_3$): δ (ppm), 7.37–7.31 (m, 4H), 7.25–7.24 (m, 1H), 4.99 (d, *J* = 10.8Hz, 1H), 4.54 (t, *J* = 6Hz, 1H) , 4.47 (d, *J* = 10.8H, 1H), 4.01–3.96 (m, 1H), 3.57–3.52 (m, 1H), 3.30–3.20 (m, 2H), 2.95-2.82 (m, 3H), 2.78–2.72 (m, 1H). ^{13}C NMR (150 MHz, $CDCl_3$): δ (ppm), 155.60, 154.76, 154.67, 152.78, 147.36, 147.32, 146.45, 146.42, 146.33, 146.32, 146.24, 146.24, 146.21, 146.19, 146.16, 146.16, 145.98, 145.73, 145.67, 145.63, 145.55, 145.53, 145.50, 145.46, 145.40, 144.77, 144.64, 144.56, 144.53, 143.38, 143.27, 142.85, 142.83, 142.82, 142.32, 142.28, 142.24, 142.19, 142.00, 141.97, 141.39, 140.45, 140.43, 140.15, 139.98, 137.09, 136.26, 135.51, 135.46, 128.87, 128.70, 126.51, 118.73, 76.54, 76.31, 71.78, 66.46, 48.42, 33.63, 33.51. APCI-MS: m/z calcd for $C_{73}H_{16}N_2$: 920.1 $[M]^-$, found: 920.6.

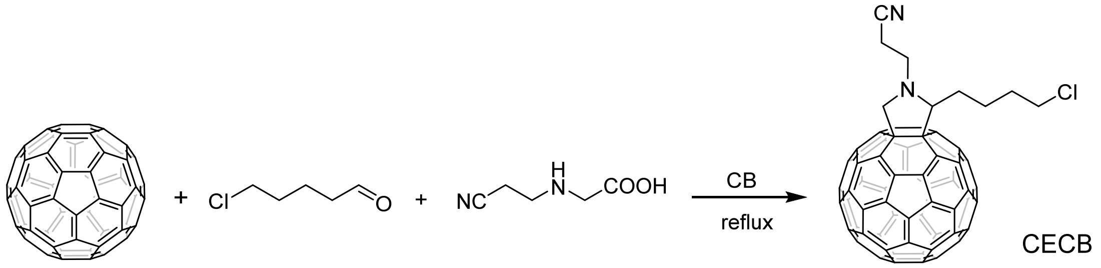

Scheme S3. Synthesis of the compound CECB.

Synthesis of compound *N*-(2-cyanoethyl)-2-(4-chlorobutyl) [60]fulleropyrrolidine (CECB). A mixture of C_{60} (0.05 mmol, 36 mg), 5-chloropentanal (0.1 mmol, 10.5 μL), *N*-(2-cyanoethyl) glycine (0.2 mmol, 32 mg) was dissolved in anhydrous chlorobenzene (10 mL) under the inert gas protection. The reaction mixture was stirred at reflux for 1.5 h (detected by TLC). The crude product was concentrated in vacuo and purified on a chromatographic column using toluene as the eluent to afford the desired product of CECB. After precipitation with methanol and drying, the brown solid of CECB was obtained with an isolated yield of 39.8% (18.0 mg). ^{1}H NMR (500 MHz, $CS_2/CDCl_3$): δ (ppm), 4.99 (d, *J* = 11Hz, 1H), 4.48 (t, *J* = 6.5Hz, 1H), 4.44 (d, *J* = 10.5Hz, 1H), 3.99–3.94(m, 1H), 3.63(t, *J* = 6.5Hz, 2H), 3.54–3.49(m, 1H), 2.93(t, *J* = 6.5Hz, 2H), 2.59–2.44(m, 2H), 2.18–2.05(m, 2H), 2.04–1.95(m, 2H). ^{13}C NMR (125 MHz, $CS_2/CDCl_3$): δ (ppm), 155.33, 154.32, 154.30, 152.46, 147.10, 147.07, 146.23, 146.20, 146.11, 146.09, 145.99, 145.96, 145.93, 145.74, 145.50, 145.40, 145.38, 145.37, 145.35, 145.32, 145.30, 145.21, 145.15, 144.56, 144.41, 144.32, 144.29, 143.16, 143.06, 142.65, 142.63, 142.61, 142.08, 142.07, 142.05, 142.04, 142.02, 141.98, 141.78, 141.73, 140.28, 140.26, 140.02, 139.77, 136.87, 136.03, 135.34, 135.26, 117.60, 76.94, 76.25, 71.35, 66.53, 48.56, 44.52, 32.99, 31.21, 24.80, 18.58. APCI-MS: m/z calcd for $C_{69}H_{15}ClN_2$: 906.1 $[M]^-$, found: 905.9.

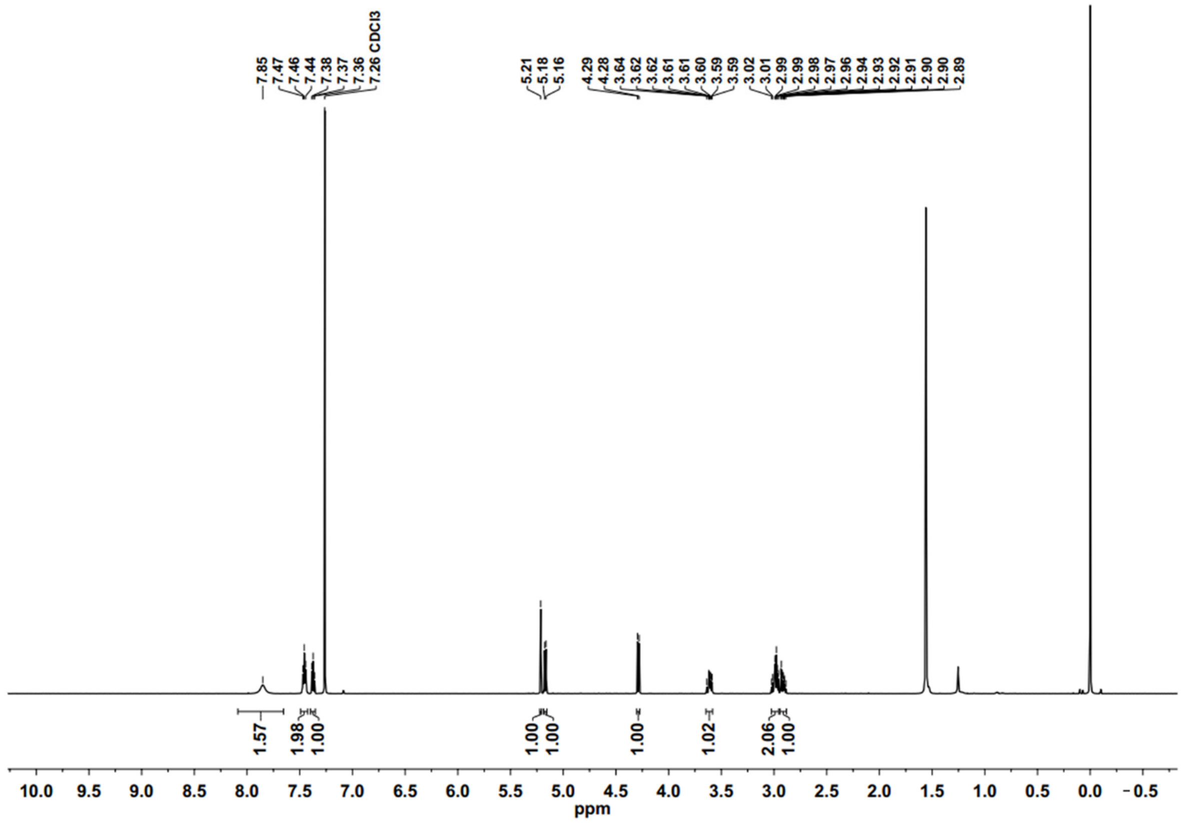

Figure S1. ^1H NMR spectrum (600 MHz) of compound CEP with $CDCl_3$ as the internal lock.

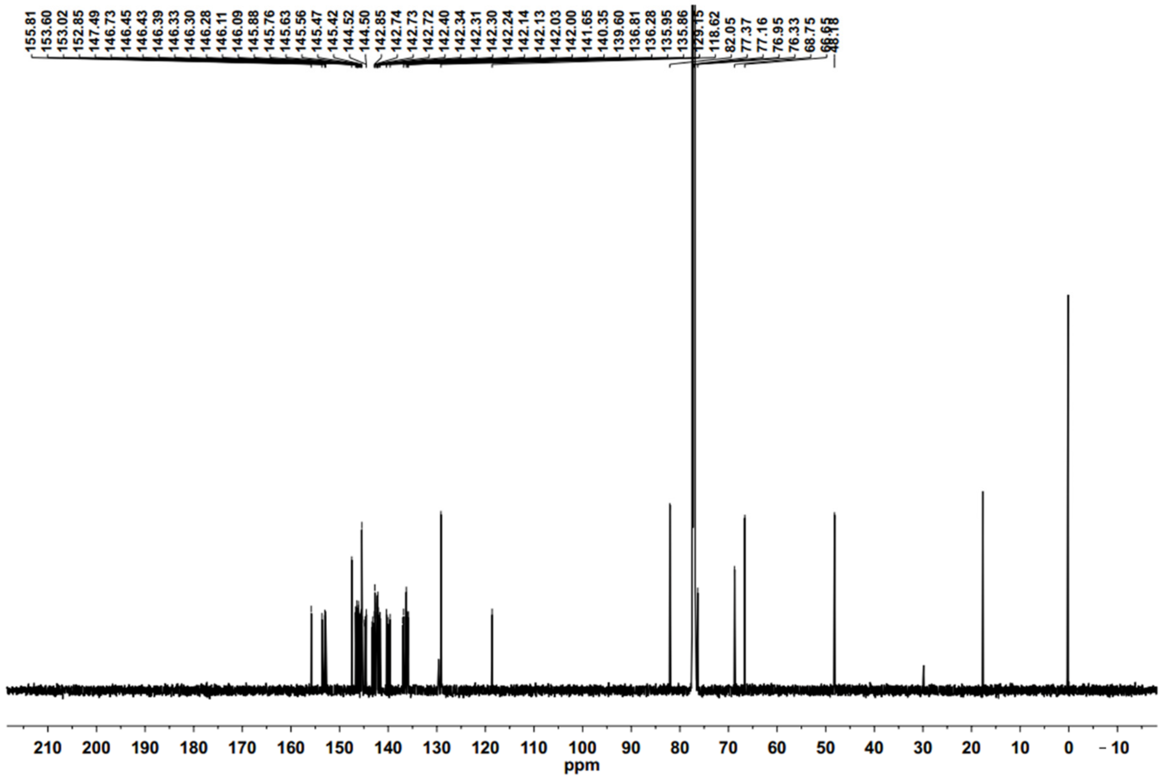

Figure S2. ^{13}C NMR spectrum (150 MHz) of compound CEP with $CDCl_3$ as the internal lock.

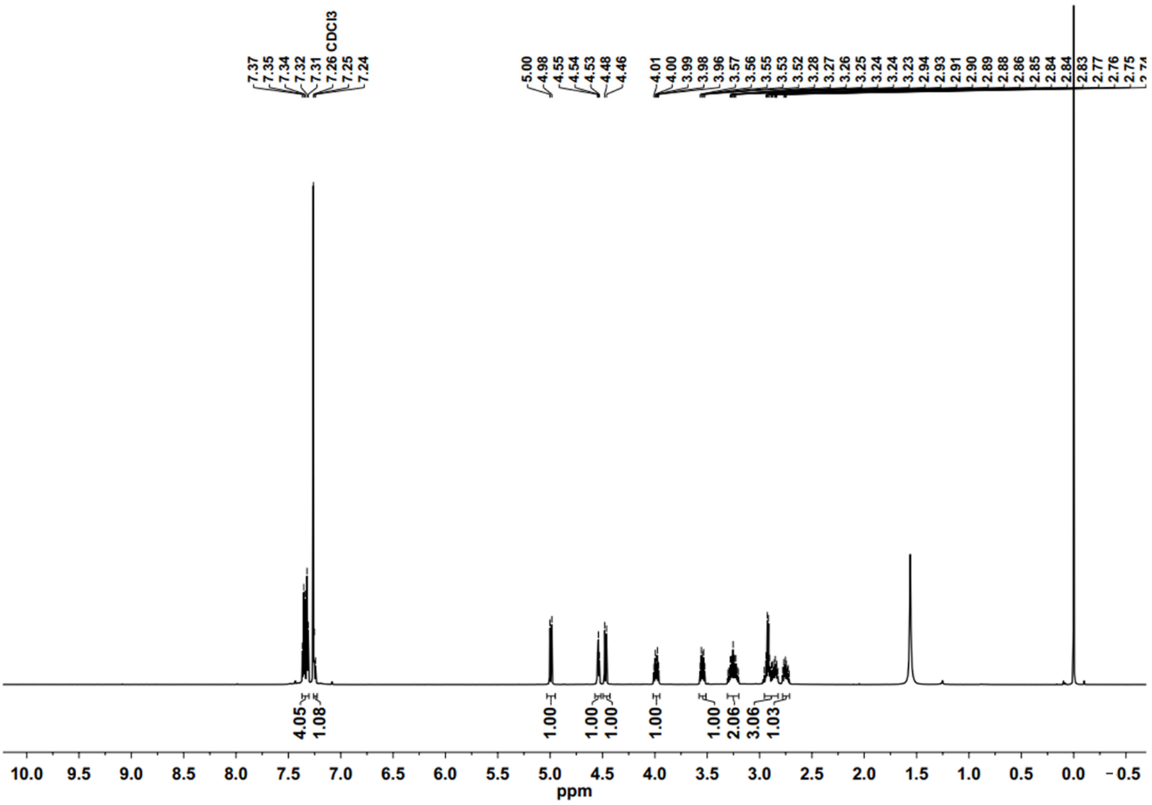

Figure S3. ^{1}H NMR spectrum (600 MHz) of compound CEPE with $CDCl_3$ as the internal lock.

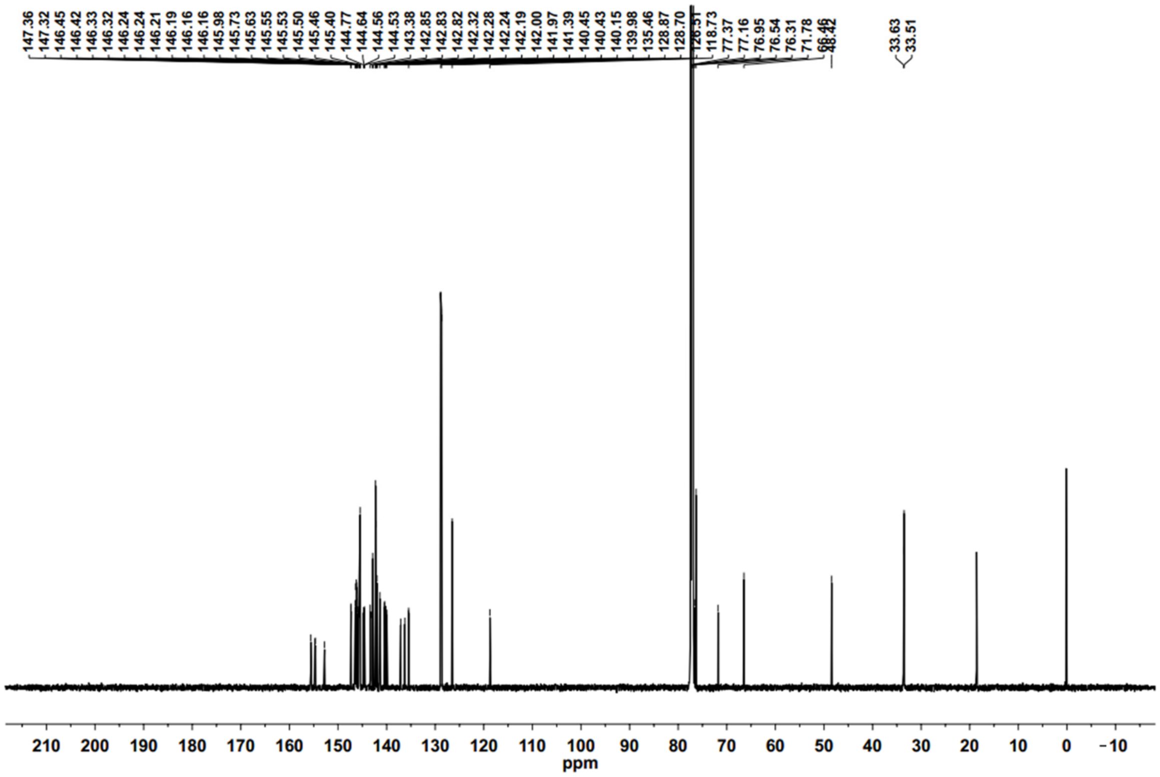

Figure S4. ^{13}C NMR spectrum (150 MHz) of compound CEPE with $CDCl_3$ as the internal lock.

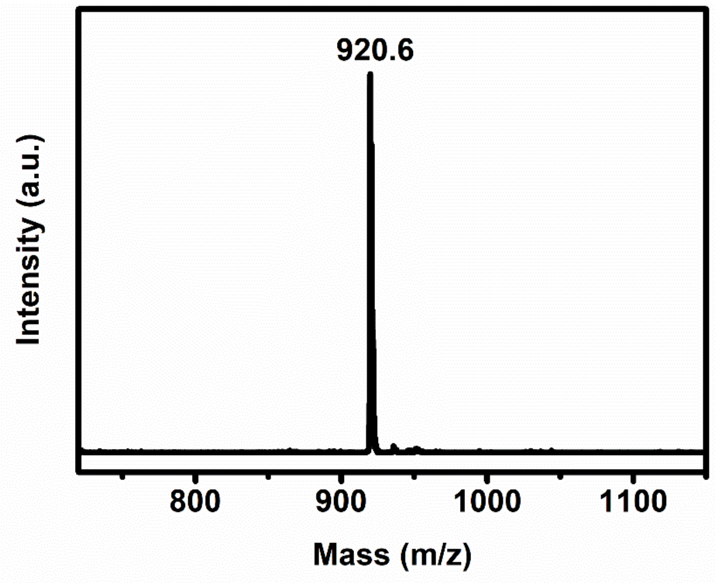

Figure S5. APCI-MS spectrum of compound CEPE conducted in the mode of negative ions.

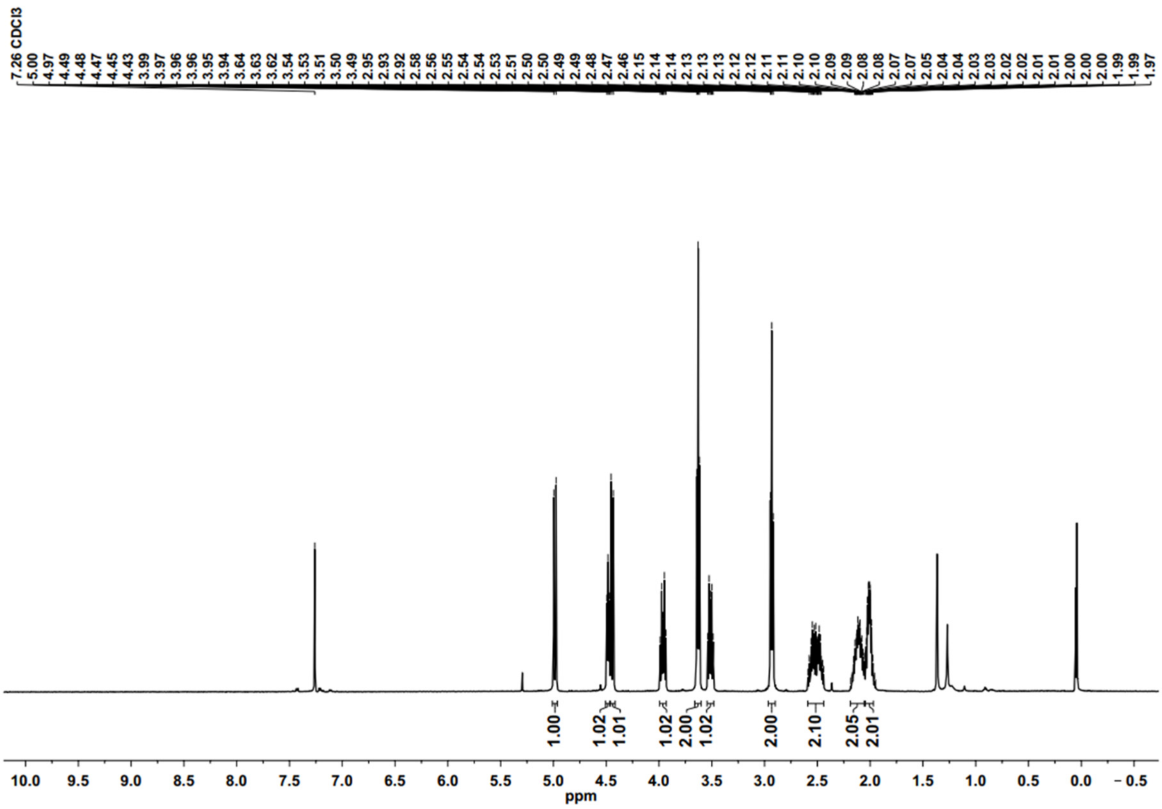

Figure S6. ^{1}H NMR spectrum (500 MHz) of compound CECB in CS_2 with $CDCl_3$ as the internal lock.

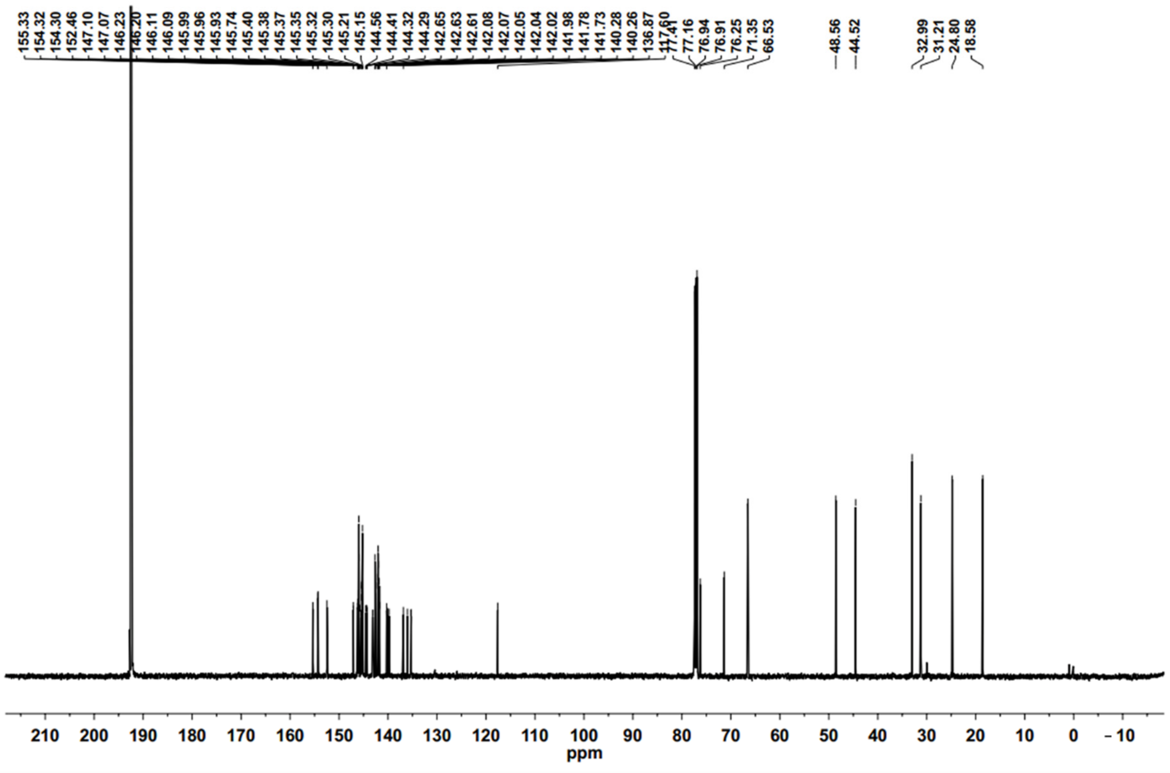

Figure S7. ^{13}C NMR spectrum (125 MHz) of compound CECB in CS_2 with $CDCl_3$ as the internal lock.

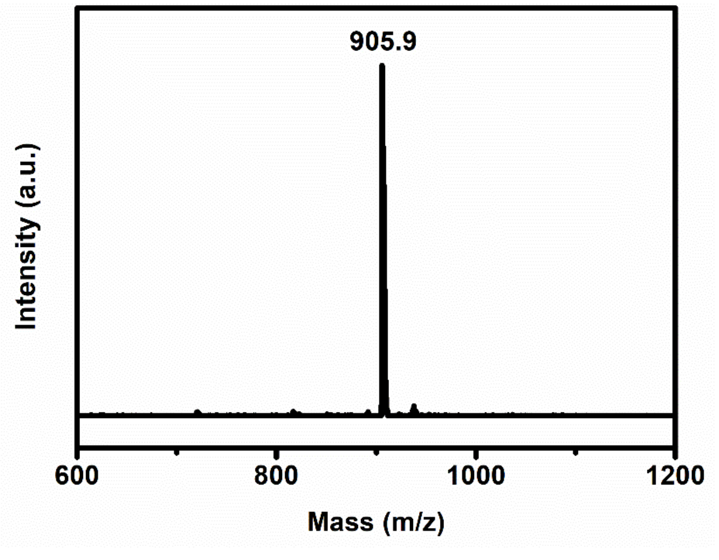

Figure S8. APCI-MS spectrum of compound CECB conducted in the mode of negative ions.

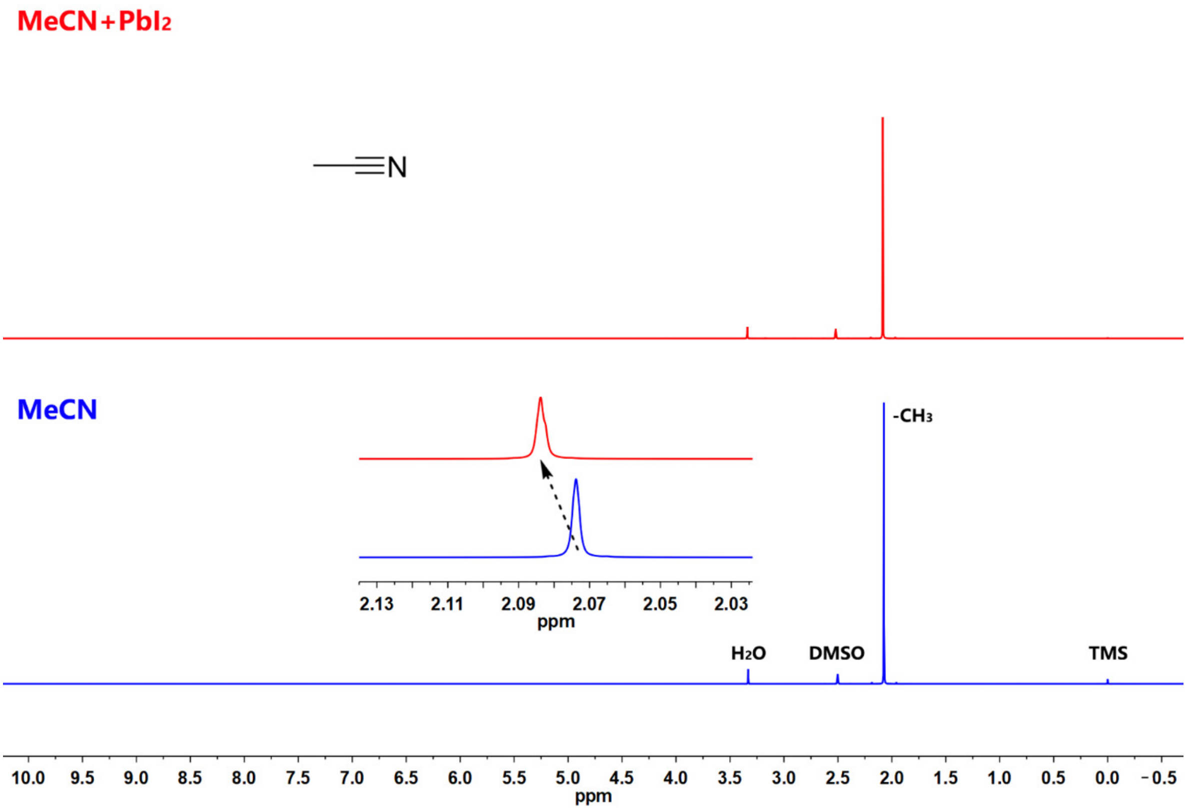

Figure S9. The comparison of NMR spectra between MeCN and the mixture of MeCN + PbI_2 in DMSO-d_6 contains 0.03% (v/v) TMS. The peak at 0.00 ppm was attributed to TMS which was an inner reference for the measurement.

The comparison of liquid state ^1H NMR spectra between MeCN and mixture of MeCN + PbI_2 are shown in Figure S9. With the addition of Pb^{2+} to the solution of MeCN, protons H on the methyl moieties display a slight downfield shift. The variation of chemical shift indicate the interaction between -CN and Pb^{2+}. [2]

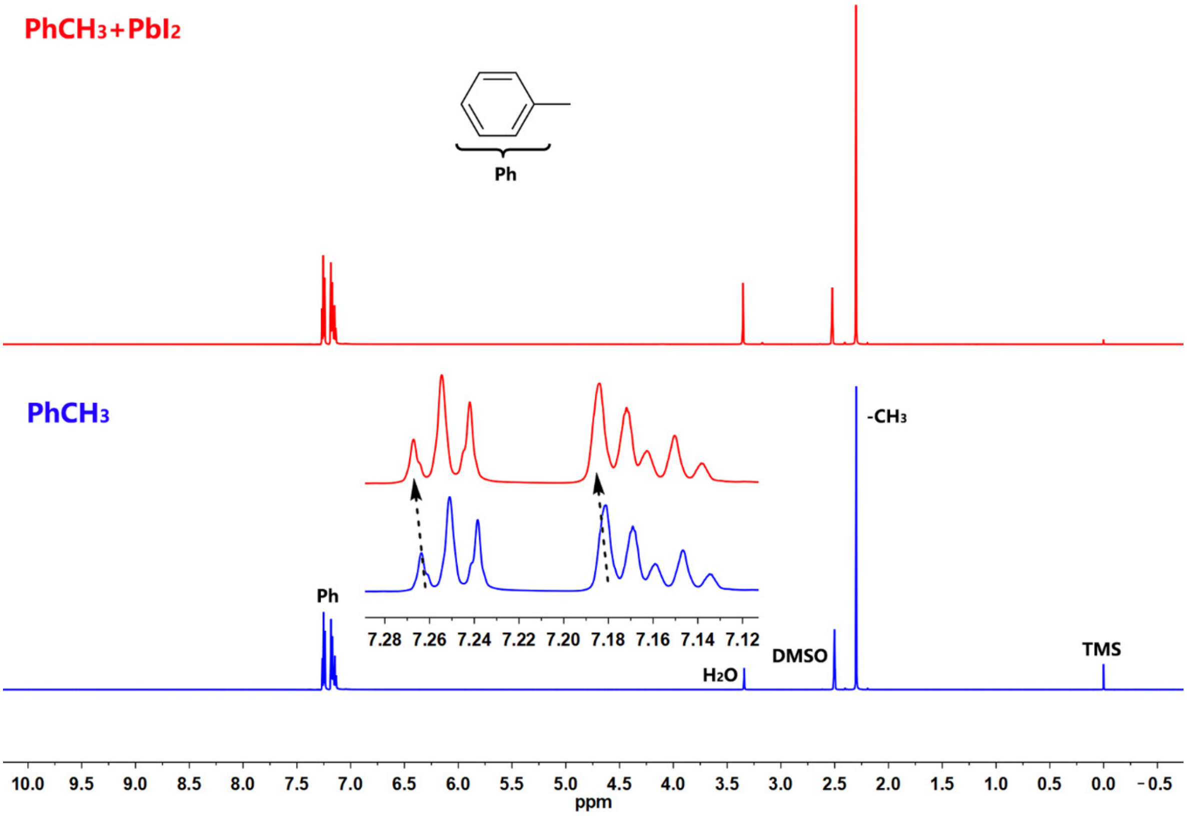

Figure S10. The comparison of NMR spectra between $PhCH_3$ and the mixture of $PhCH_3$ + PbI_2 in DMSO-d_6 contains 0.03% (v/v) TMS. The peak at 0.00 ppm was attributed to TMS which was an inner reference for the measurement.

The comparison of liquid state ^1H NMR spectra between $PhCH_3$ and the mixture of $PhCH_3$ + PbI_2 are shown in FigureS10. With the addition of Pb^{2+} to the solution of $PhCH_3$, protons H on the phenyl moieties display a slight downfield shift, while protons H on the methyl moieties display no shifts. These differences on chemical shift are attributed to the cation–π interaction between Pb^{2+} and π-conjugated benzene ring structure. [3]

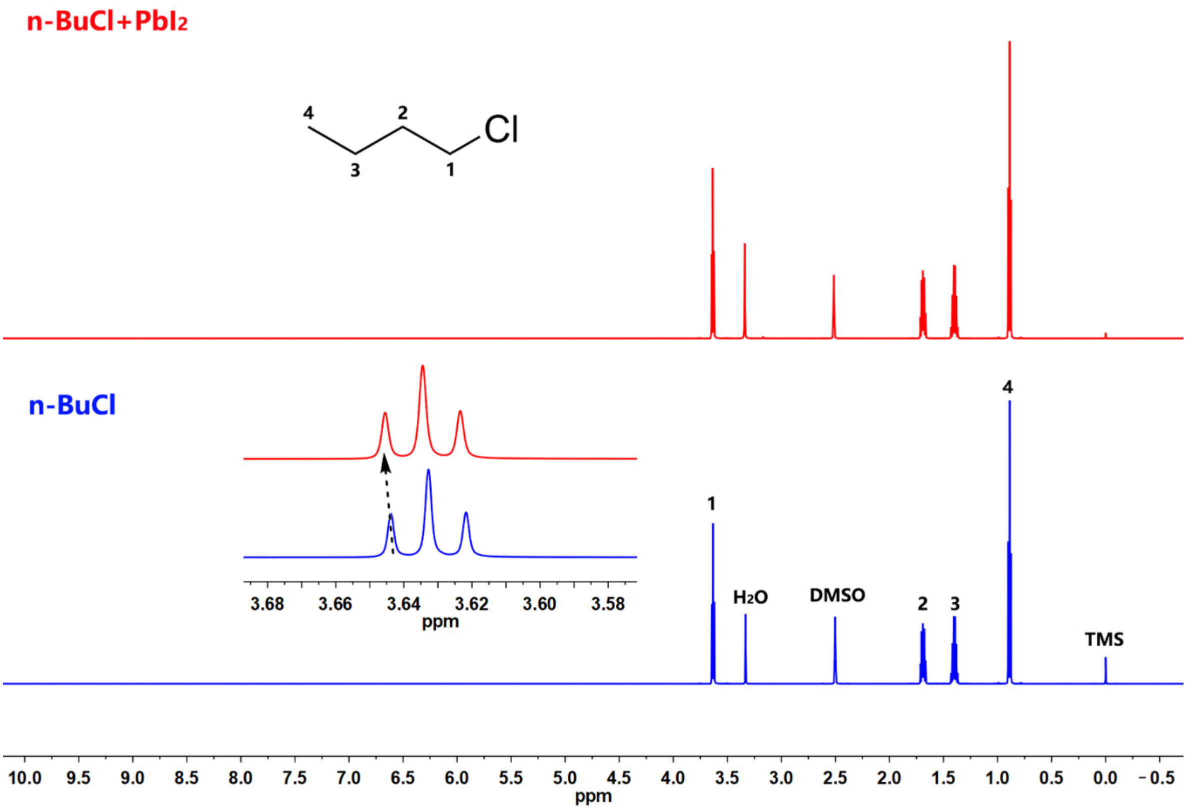

Figure 11. The comparison of NMR spectra between *n*-BuCl and the mixture of *n*-BuCl + PbI_2 in DMSO-d_6 contains 0.03% (v/v) TMS. The peak at 0.00 ppm was attributed to TMS which was an inner reference for the measurement.

The comparison of liquid state ^1H NMR spectra between *n*-BuCl and the mixture of *n*-BuCl + PbI_2 are shown in Figure S11. Different peaks corresponding to different ^1H are clearly marked. With the addition of Pb^{2+} to the solution of *n*-BuCl, protons H marked "1" adjacent to Cl atom display a slight downfield shift, and other protons H on the alkyl chain display no shifts. These different chemical shifts are attributed to the influence of the interaction between Pb^{2+} and Cl. [4]

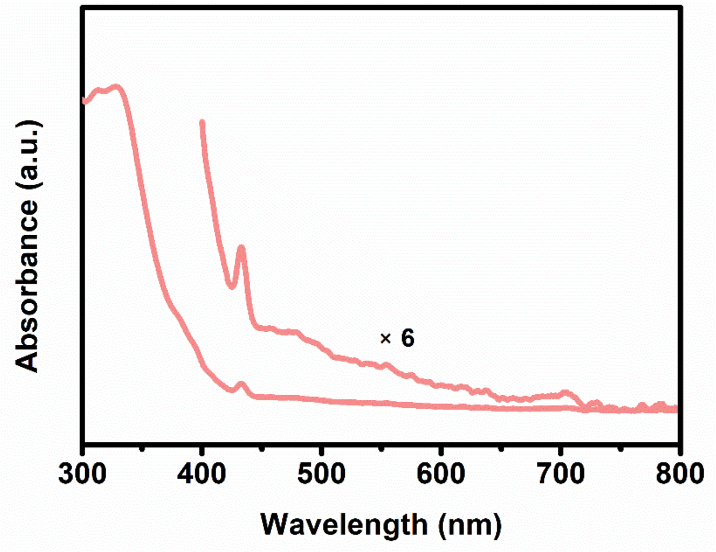

Figure S12. UV-Vis absorption spectra of CEP in toluene solution.

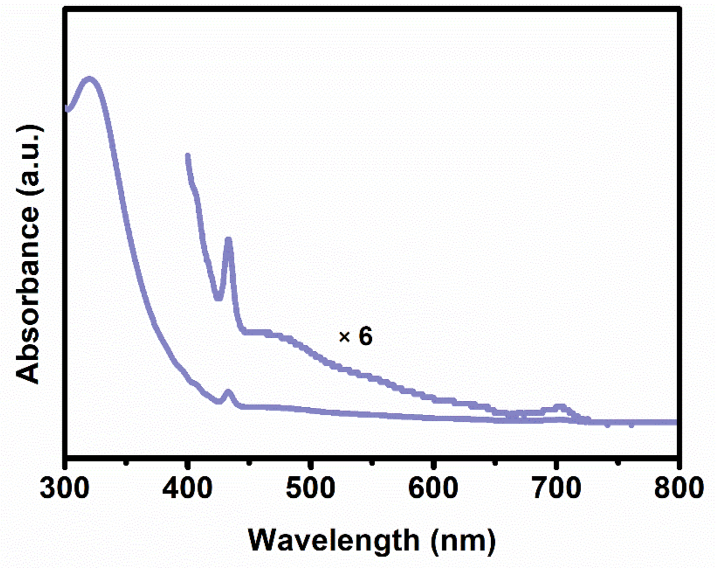

Figure S13. UV-Vis absorption spectra of CEPE in toluene solution.

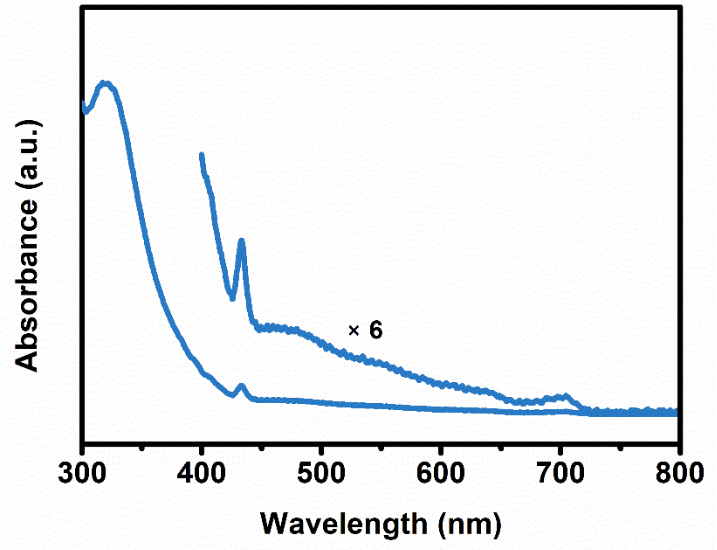

Figure S14. UV-Vis absorption spectra of CECB in toluene solution.

Table S1. Electrochemical and optical properties of CEP, CEPE and CECB.

Compound	$E_{o,\,red}$ (V)[a]	LUMO (eV)[b]	$\lambda_{o,\,max}$ (nm)	E_g (eV)	HOMO (eV)
CEP	−1.06	−3.74	718	1.73	−5.47
CEPE	−1.06	−3.74	718	1.73	−5.47
CECB	−1.05	−3.75	718	1.73	−5.48

[a] **Values are versus Fc/Fc$^+$.**

[b] **Estimated using the following equation: LUMO = −e ($E_{o,\,red}$ + 4.8)** (eV), where $E_{o,\,red}$ is the onset reduction potential in volt *vs* Fc/Fc$^+$. [5].

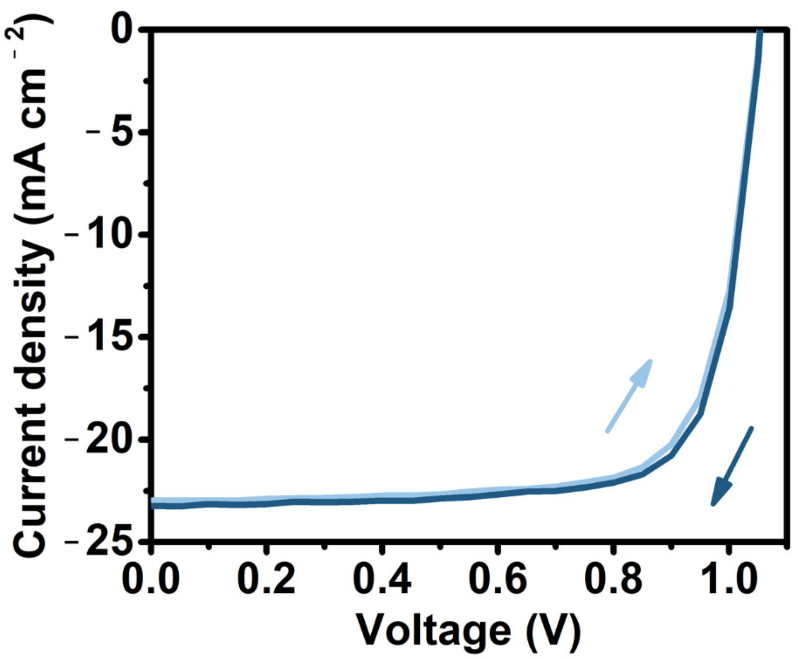

Figure S15. *J-V* curves of CECB-based device under different scan directions.

Table S2. Photovoltaic performance and stability of fulleropyrrolidine-based PSCs in the literature.

Device Structure	PCE (%)	Stability	Ref.
FTO/PEDOT:PSS/Perovskite/**DMEC$_{70}$**/LiF/Al	16.4	10 days, RT, air	[6]
ITO/PEDOT:PSS/Perovskite/**C$_{60}$-N**/Al	16.6	200 hours, RT, air	[7]
ITO/**CPTA**/Perovskite/Spiro-OMeTAD/Au	18.4	100 days, RT, N_2	[8]
ITO/PEDOT:PSS/Perovskite/**α-DMEC$_{70}$**/Al	18.6	10 days, RT, air	[9]
FTO/NiO_x/Perovskite/**C$_{60}$-BPy**/BCP/Ag	16.8	1600 hours, RT, air	[10]
ITO/P3CT/Perovskite/**CPTA-E**/Al	17.4	7 days, RT, air	[11]
FTO/**PTEG-1**/Perovskite/Spiro-OMeTAD/Au	16.2	1000 hours, 60°C, air	[12]
ITO/P3CT-Na/Perovskite/**PDI-C$_{60}$**/BCP/Ag	18.6	500 hours, RT, air	[13]
FTO/NiO_x/Perovskite/**C$_{60}$-3-Py**/BCP/Ag	17.6	1400 hours, RT, air	[14]
ITO/NiO_x/PTAA/Perovskite/**F4**/BCP/Ag	20.4	1056 hours, RT, air	[1]
ITO/PTAA/Perovskite/**CECB**/BCP/Ag	**19.1**	**1800 hours**, RT, N_2	**This work**

Table S3. Photovoltaic performance of 20 devices based on different fullerene ETMs.

ETM	V_{oc} (V)	J_{sc} (mA cm^{-2})	FF (%)	PCE (%)	R_s* (Ω cm^2)	R_{sh}* (Ω cm^2)
CEPE	1.05±0.01 (1.05)*	22.94±0.20 (22.94)*	73.54±1.45 (74.81)*	17.95±0.40 (18.33)*	1.55	2153.39
CEP	1.05±0.01 (1.05)*	23.05±0.34 (23.04)*	75.27±0.65 (76.85)*	18.21±0.28 (18.68)*	1.52	3588.13
CECB	1.05±0.01 (1.05)*	23.33±0.41 (23.55)*	75.72±0.79 (77.01)*	18.36±0.42 (19.05)*	1.43	5284.94

*** Photovoltaic parameters of the champion device recorded by source meter.**

References

1. Xing, Z.; Liu, F.; Li, S.-H.; Chen, Z.-C.; An, M.-W.; Zheng, S.; Jen, A.K.Y.; Yang, S. Multifunctional molecular design of a new fulleropyrrolidine electron transport material family engenders high performance of perovskite solar cells. *Adv. Funct. Mater.* **2021**, *31*, 2107695, doi:10.1002/adfm.202107695.
2. Liu, K.; Dai, S.; Meng, F.; Shi, J.; Li, Y.; Wu, J.; Meng, Q.; Zhan, X. Fluorinated fused nonacyclic interfacial materials for efficient and stable perovskite solar cells. *J. Mater. Chem. A* **2017**, *5*, 21414-21421, doi:10.1039/C7TA06923E.
3. Wei, D.; Ma, F.; Wang, R.; Dou, S.; Cui, P.; Huang, H.; Ji, J.; Jia, E.; Jia, X.; Sajid, S.; et al. Ion-migration inhibition by the cation–π Interaction in perovskite materials for efficient and stable perovskite solar cells. *Adv. Mater.* **2018**, *30*, 1707583, doi:10.1002/adma.201707583.
4. Tan, H.; Jain, A.; Voznyy, O.; Lan, X.; García de Arquer, F.P.; Fan James, Z.; Quintero-Bermudez, R.; Yuan, M.; Zhang, B.; Zhao, Y.; et al. Efficient and stable solution-processed planar perovskite solar cells via contact passivation. *Science* **2017**, *355*, 722-726, doi:10.1126/science.aai9081.
5. Zhan, X.-X.; Zhang, X.; Dai, S.-M.; Li, S.-H.; Lu, X.-Z.; Deng, L.-L.; Xie, S.-Y.; Huang, R.-B.; Zheng, L.-S. Tailorable $PC_{71}BM$ isomers: using the most prevalent electron acceptor to obtain high-performance polymer solar cells. *Chem. Eur. J.* **2016**, *22*, 18709-18713, doi:10.1002/chem.201604263.
6. Tian, C.; Castro, E.; Wang, T.; Betancourt-Solis, G.; Rodriguez, G.; Echegoyen, L. Improved performance and stability of inverted planar perovskite solar cells using fulleropyrrolidine layers. *ACS Appl. Mater. Interfaces* **2016**, *8*, 31426-31432, doi:10.1021/acsami.6b10668.
7. Li, Y.; Lu, K.; Ling, X.; Yuan, J.; Shi, G.; Ding, G.; Sun, J.; Shi, S.; Gong, X.; Ma, W. High performance planar-heterojunction perovskite solar cells using amino-based fulleropyrrolidine as the electron transporting material. *J. Mater. Chem. A* **2016**, *4*, 10130-10134, doi:10.1039/C6TA03284B.
8. Wang, Y.-C.; Li, X.; Zhu, L.; Liu, X.; Zhang, W.; Fang, J. Efficient and hysteresis-free perovskite solar cells based on a solution processable polar fullerene electron transport layer. *Adv. Energy Mater.* **2017**, *7*, 1701144, doi:10.1002/aenm.201701144.
9. Castro, E.; Zavala, G.; Seetharaman, S.; D'Souza, F.; Echegoyen, L. Impact of fullerene derivative isomeric purity on the performance of inverted planar perovskite solar cells. *J. Mater. Chem. A* **2017**, *5*, 19485-19490, doi:10.1039/C7TA06338E.
10. Li, B.; Zhen, J.; Wan, Y.; Lei, X.; Liu, Q.; Liu, Y.; Jia, L.; Wu, X.; Zeng, H.; Zhang, W.; et al. Anchoring fullerene onto perovskite film via grafting pyridine toward enhanced electron transport in high-efficiency solar cells. *ACS Appl. Mater. Interfaces* **2018**, *10*, 32471-32482, doi:10.1021/acsami.8b11459.
11. Chang, J.; Wang, Y.-C.; Song, C.; Zhu, L.; Guo, Q.; Fang, J. Carboxylic ester-terminated fulleropyrrolidine as an efficient electron transport material for inverted perovskite solar cells. *J. Mater. Chem. C* **2018**, *6*, 6982-6987, doi:10.1039/C8TC01955J.
12. Liu, X.; Li, P.; Zhang, Y.; Hu, X.; Duan, Y.; Li, F.; Li, D.; Shao, G.; Song, Y. High-efficiency perovskite solar cells based on self-assembly n-doped fullerene derivative with excellent thermal stability. *J. Power Sources* **2019**, *413*, 459-466, doi:10.1016/j.jpowsour.2018.12.066.
13. Luo, Z.; Wu, F.; Zhang, T.; Zeng, X.; Xiao, Y.; Liu, T.; Zhong, C.; Lu, X.; Zhu, L.; Yang, S.; et al. Designing a perylene diimide/fullerene hybrid as effective electron transporting material in inverted perovskite solar cells with enhanced efficiency and stability. *Angew. Chem. Int. Ed.* **2019**, *58*, 8520-8525, doi:10.1002/anie.201904195.
14. Li, B.; Zhen, J.; Wan, Y.; Lei, X.; Jia, L.; Wu, X.; Zeng, H.; Chen, M.; Wang, G.-W.; Yang, S. Steering the electron transport properties of pyridine-functionalized fullerene derivatives in inverted perovskite solar cells: the nitrogen site matters. *J. Mater. Chem. A* **2020**, *8*, 3872-3881, doi:10.1039/C9TA12188A.
